# Correction osteotomy of distal radius malunion stabilised with dorsal locking plates without grafting

**DOI:** 10.1007/s11751-014-0190-2

**Published:** 2014-03-08

**Authors:** D. Tiren, D. I. Vos

**Affiliations:** Department of General and Trauma Surgery, Amphia Hospital, Postbus 90158, 4800 RK Breda, The Netherlands

**Keywords:** Correction osteotomy, Corrective osteotomy, Distal radius, Malunion, Bone graft, Locking plates

## Abstract

The purpose of this study was to evaluate the results of our correction osteotomies of distal radial malunions without a bone graft. Eleven consecutive patients (mean age 52 years, range 18–71) were treated. A dorsal approach was utilised to perform an opening-wedge osteotomy which then was stabilised with two dorsal columnar plates without filling the osteotomy gap. All patients went on to radiographic union with a filling of the osteotomy gap within a mean period of 3 months (range 2–6 months). All patients had satisfactory results in terms of function and pain. Correction osteotomy and stabilisation with bicolumnar locked plate fixation without a bone graft provides sufficient stability to allow the highly vascularised metaphysis to heal. In patients without risk factors predisposing to non-union, this procedure is safe and feasible.

## Introduction

The distal radius fracture is the most common fracture treated in the emergency department. The most common complication of this fracture is malunion which occurs in up to 25 % of conservatively and in 10 % of operatively treated cases [1]. Although a small part of these fractures lead to a symptomatic malunion, correction osteotomy is a frequently performed procedure due to the high incidence of the distal radius fracture [2].

Opening-wedge correction osteotomy of the distal radius is an established but challenging procedure [3]. By this procedure, the foremost complaints such as limited wrist and forearm motion and reduced grip strength are improved and pain diminishes as reported by over 200 papers [3]. The outcome of the procedure is determined by patient selection and adequate restoration of the original anatomy [1, 4, 5].

The procedure consists of correcting the anatomy by performing an osteotomy, filling of the osteotomy gap while maintaining the correction, and stabilising it with plates. Traditionally, the osteotomy gap is filled with an iliac crest corticocancellous bone graft and supported by rigid plate fixation. Although use of other donor sites has been described, the iliac crest remains the golden standard. The harvesting of iliac crest bone can result in donor-site morbidity as high as 20 % [6]. The alternative is to use expensive bone graft substitutes, while the optimal replacement material for the distal radius remains unclear [7].

By leaving the osteotomy gap unfilled, the additional procedure of harvesting the graft is avoided and the surgeon can concentrate on the most important aspect of the procedure that determines the outcome, which is correction of the anatomical deformity.

In this paper, we describe the results of distal radius correction osteotomy in patients with symptomatic malunion with dorsal bicolumnar locked plating, without filling the osteotomy defect.

## Patients and methods

### Patient selection

Between 2009 and 2011, we treated eleven consecutive patients with a symptomatic dorsal malunion of the distal radius. The medical files, radiographs and outpatient notes were retrospectively analysed.

All patients had undergone an opening-wedge osteotomy through a dorsal approach and dorsal bicolumnar locked plating with 2.4 mm columnar stabilisation plates from Synthes©. The osteotomy gap was left unfilled.

The patient demographics, fracture characteristics and initial treatment method along with the symptoms are summarised in Table 1.

**Table 1:** Patient demographics, fracture characteristics, initial treatment and remarks

Patient Nr/sex/age	AO/Fernandez classification	Delay of osteotomy (weeks)	Fracture side/dominance	Foremost complaint at presentation	Initial treatment	Additional remarks
1/M/18	C1/III	19	R/R	Pain in rest and movement	Cast immobilisation	–
2/M/63	C1/III	23	L/R	Functional impairment	Cast immobilisation	–
3/M/62	A3/I	14	L/R	Pain in rest and movement	Cast immobilisation	–
4/F/41	C1/III	19	R/R	Pain in rest and movement	Mini AO double plates	Contralateral distal radial fracture
5/F/36	A2/I	13	L/R	Pain in rest and impaired strength	Cast immobilisation	Plates removed at 6 months
6/F/72	A2/I	16	L/R	Pain and functional impairment	Cast immobilisation	CRPS-1
7/F/57	A3/I	26	L/R	Pain and impaired strength	Cast immobilisation	TFCC tear
8/F/49	A2/I	21	R/R	Pain and carpal tunnel compression	Cast immobilisation	CTS
9/M/47	C1/III	15	L/R	Pain and functional impairment	Cast immobilisation	–
10/F/61	A2/I	37	L/R	Carpal tunnel compression and functional impairment	Cast immobilisation	CTS
11/F/66	A2/I	11	L/L	Pain, functional impairment	External fixation	–

*M* male, *F* female, *L* left, *R* right, *TFCC* triangular fibrocartilaginous complex, *CRPS-1* complex regional pain syndrome type 1, *CTS* carpal tunnel syndrome

The indication for correction osteotomy of the distal radius was based mainly on clinical findings such as pain and functional impairment in combination with findings on X-ray. A dorsal angulation of more than 15° or a substantial dorsal angulation with a more than 2 mm shortening of the radial height were the accompanying radiological criteria mostly used. The preoperative workup included a CT scan of the malunited fracture. On indication, an MRI and routinely an X-ray of the contralateral wrist to determine the normal anatomical relationship were performed.

Patients with risk factors known to predispose to non-union of the distal radius, such as morbid obesity, tobacco abuse and diabetes mellitus were treated by other means.

### Surgical technique

The dorsal approach to the distal radius was utilised as described by Rikli and Regazzoni [8]. The procedure was carried out under general or regional anaesthesia with a tourniquet and fluoroscopic control. The forearm and hand were disinfected and draped. A 7–10-cm straight incision was performed, over an imaginary line between the base of the second metacarpal, crossing over Listers tubercle towards the border of the muscle belly of the first extensor compartment. For our purpose, the part of the incision distal to the joint crease was not utilised. The subcutaneous tissue was divided, and the extensor retinaculum was incised through the third compartment to identify and free the extensor pollicis longus (EPL) tendon. It was held aside with a vessel loop and sometimes behind a Hohmann or a wound retractor. The floor of the third compartment was incised and subperiostally dissected to preserve the second and the fourth compartments. Radially dissection was towards the brachioradialis tendon and ulnarly towards the distal radioulnar joint.

The correction osteotomy was performed as described by Fernandez [4]. Approximately 2 centimetres proximal to the wrist joint the osteotomy site was marked and two K-wires placed; one parallel to the wrist joint and the other tangential to the radius. The osteotomy was performed with an oscillating saw, parallel to the distal K-wire. Using a laminar spreader, the osteotomy was widened until the K-wires were parallel to each other, volar cortical contact being maintained. Using preoperative measurements, additional corrections were performed to correct radial inclination by positioning the laminar spreader more radially while keeping the K-wires parallel. In some cases, an external fixator was placed between the two K-wires to support the reduction with the laminar spreader, while small adjustments were made. When the optimal reduction was achieved under fluoroscopic control, first the dorsoradial plate was positioned underneath the second compartment to support the radial styloid. Then, the radioulnar plate was placed in an angle of approximately 50°–70° to the first plate. After preliminary fixation of the plates, the laminar spreader was removed and the correction verified before definitive fixation of the plates. At least two locked screws were used proximal and distal to the osteotomy site in both plates. The EPL tendon was protected by creating a flap from the extensor tendon retinaculum. The skin was sutured, no wound drains were used, and a compressive bandage was placed over the operated area as far as the elbow.

### Postoperative treatment and follow-up

Postoperatively early finger motion and forearm rotation were encouraged. A removable splint was provided for comfort for the first 2 weeks. After splint removal, the wrist was actively and passively mobilised. Powerful and resistive exercises were prohibited until radiographic healing. All other comfortable reasonable daily activities were allowed.

At 2, 6 and 12 weeks and if necessary every 6 weeks after 3 months, patients visited the outpatient clinic where the range of movement of the wrist was assessed and X-rays obtained. Patients were discharged after radiological union and a satisfactory clinical result.

### Data collection and statistical analysis

Preoperatively informed consent was obtained, including the disclosure of the possibility of delayed union and non-union which would require a second operation. Range of movement and radiographic changes were documented during each outpatient visit. At the final outpatient visit, the quick-DASH and the Mayo Wrist scores were evaluated. The digitally calibrated radiographs were analysed on high-resolution terminals and measurements performed on preoperative, postoperative and the final radiographs by the first author.

A gradual closing of the osteotomy gap could be observed clearly on the lateral radiographs of the distal radius (Fig. 1). This observation was used to determine union. Closure of the osteotomy gap began at the volar side and followed to the dorsal side until dorsal cortical bridging was complete. The complete dorsal bridging of the osteotomy gap was defined as the moment of union.

**Fig. 1:**
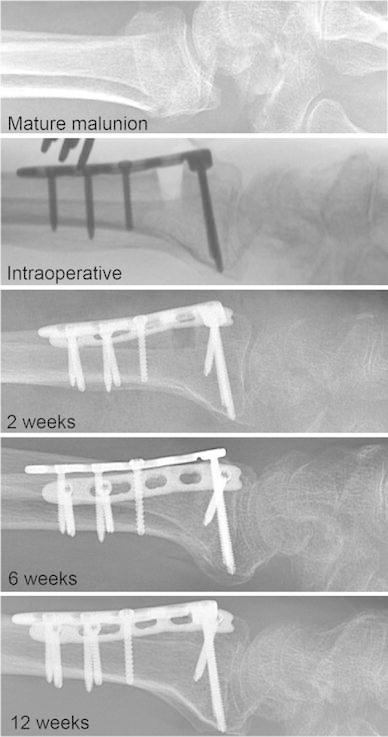
Correction of the deformity and healing of the osteotomy gap

Pre- and postoperative measurements were compared using dependent sample *t* test for continuous data. *p* values of <0.05 were considered statistically significant.

## Results

There were no preoperative or postoperative complications on follow-up evaluation (average of 8 months, range 6–13 months) of our case series. The radiological and functional findings pre- and postoperatively and the results of the outcome scoring postoperatively are summarised in Table 2.

**Table 2:** Summary of radiological and functional results

	Preoperative Mean (±SD)	Postoperative Mean (±SD)	Improvement (±SD)	*p* value Student’s *t* test
*Radiographic measurements*				
Volar tilt (°)	−22 (7)	4 (6)	26 (6)	*p* < 0.0001
Radial inclination (°)	16 (3)	22 (3)	7 (2)	*p* = 0.0002
Radial height (mm)	7 (3)	14 (2)	6 (5)	*p* < 0.0001
*Range of motion*				
Wrist flexion (°)	34 (16)	71 (13)		*p* < 0.0001
Wrist extension (°)	45 (20)	67 (15)		*p* = 0.010
Forearm pronation (°)	52 (16)	85 (6)		*p* < 0.0001
Forearm supination (°)	41 (19)	80 (7)		*p* < 0.0001
Flexion–extension arc (°)	79 (29)	138 (21)	59 (30)	*p* < 0.0001
Pronation–supination arc (°)	93 (32)	165 (12)	72 (33)	*p* < 0.0001
*Functional outcome scores*				
Mayo wrist score		92 (6)		
q-DASH score		10 (6)		
*Time to union*		13 (6)		

### Radiological findings

All osteotomies demonstrated a radiological union of the osteotomy deficit in a mean time of 13 weeks (range 6–24 weeks). There was a correlation between the degree of angular correction and the time to healing of the osteotomy gap (Fig. 2).

**Fig. 2:**
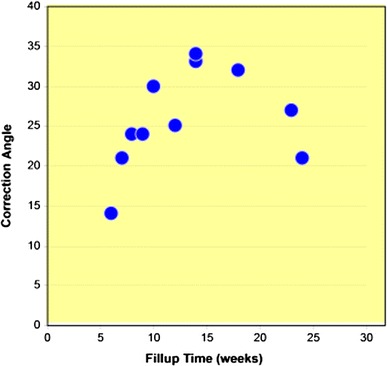
Correlation between the correction angle and the fill-up time

The dorsal tilt was corrected by the procedure with a mean of 26°, from a mean dorsal flexion of 22° before the procedure (range 30°–6° dorsal tilt) to a mean palmar flexion of 4° after the procedure (range 2°–15° volar tilt). Radial inclination was improved from a mean of 16 (range 8°–20°) to 22 (range 18°–26°) degrees by the procedure. The radial height was improved from a mean of 7 (range 3–13) mm preoperatively to a mean of 14 (range 12–18) mm postoperatively.

All radiological measurements showed statistically significant improvement postoperatively compared with preoperative measurements.

The follow-up radiographs during the different intervals did not show any loss of reduction or implant failure. Neither were osteoarthritic changes seen during this follow-up.

### Clinical outcomes

During the final follow-up visit, a mean wrist and forearm motion was achieved of 71° of flexion (range 40–85), 67° of extension (range 40–90), 85° of pronation (range 70–90) and 80° of supination (range 65–85), when compared with a mean wrist and forearm motion of 34° flexion (range 10–45), 45° of extension, 52° pronation (range 30–70) and 41° of supination (range 10–60) preoperatively. The wrist flexion–extension arc was improved by a mean of 59° (range 20°–110°), and the forearm rotation arc was improved by a mean of 72° (range 10°–135°).

All measurements postoperatively showed a statistically significant improvement.

The modified Mayo Wrist score outcome was excellent in 5 patients, good in 5 patients and satisfactory in one patient. The mean modified Mayo Wrist score was 92 points of 100 (range 80–100).

The q-DASH score was 10 at final follow-up (range 2.3–20.5).

The wrist function gradually improved during the follow-up visits. The pain present in most of the patients and the median nerve compression symptoms in two patients, resolved with the correction.

The TFCC tear of one patient was repaired through a separate incision. After the operation, she had a stable wrist and a good function. All patients were pain-free in rest and could perform their daily activities without pain. One of the patients had some discomfort with forceful forearm rotation during the final visit which did not limit his daily activities.

Only one patient requested removal of the osteosynthesis because of a palpable plate below the scar and a wish for correction of the scar. This procedure was performed 6 months after the correction osteotomy and we could verify the consistency of the new filled up bone which was as solid as the other parts of the distal radius.

We did not encounter complications due to the plate on the dorsal side such as tendon ruptures, tendinitis or adhesions during the follow-up period of this study.

## Discussion

We documented good results in our series where we performed a distal radial correction osteotomy for distal radial malunion stabilising it using double-locked plates without filling the osteotomy gap.

The patients had a mean correction of 26° volar tilt and 6 millimetres of radial height resulting in a mean wrist motion of 138° and a forearm rotation of 165°. We had no loss of reduction before healing of the osteotomy gap which occurred after a mean time of 13 weeks. There was no case of infection, implant failure, loss of reduction, delayed union or non-union. However, when the correction of the angle was greater and consequently the osteotomy gap wider, the time for complete union was longer. Even for duration of 24 weeks, the double-dorsal-locked plates construction proved stable enough to maintain the reduction. These results compare with and in some cases are more favourable to other published series [4, 9–11].

As described by Fernandez [4], the traditional correction osteotomy includes correction of the deformity and filling of the opened wedge with a full-thickness iliac crest bone graft. The graft should make a perfect fit with the osteotomy gap to support the reduction. This is held in place with an osteosynthesis. The function of this full-thickness corticocancellous bone graft is twofold: support of the correction and induction of bone healing.

Ring et al. [10] demonstrated that the supportive function of the corticocancellous graft is not necessary when locked plates are used. However, he used cancellous bone graft to induce bone healing in the osteotomy gap. Since cancellous graft harvesting has significant less donor-site morbidity, augmenting the osteotomy gap with cancellous graft has replaced grafting the gap with full-thickness corticocancellous bone graft when used in combination with locked plates in this procedure.

The distal radial metaphysis is a highly vascularised bone with excellent healing capacity [12]. This is why in our opinion there is no reason to induce bone healing by bone grafting, since performing the osteotomy initiates bone healing. Adding cancellous bone or any other biomaterials does not improve fracture healing in distal radial fractures [7, 13], supporting the concept of excellent healing capacity of the distal radial metaphysis.

However, several premorbid conditions seem to predispose to developing a non-union [12]. These conditions include diabetes mellitus, morbid obesity, tobacco abuse and severe peripheral vascular disease. These can be seen as the biological disturbance that affects bone healing.

Even though mechanical support of the correction after osteotomy is achieved well by the double plate construct, this mechanical stability relies on support of the volar cortex. All our osteotomies had volar contact after the procedure. In cases where lengthening is necessary, a circular defect could be created. In our opinion, this would lead to insufficient mechanical support and stability at the osteotomy site. In such cases, we would advise harvesting a full-thickness corticocancellous graft for mechanical support of the defect.

Another area, similar to the distal radius in terms of healing capacity and in being a donor site for cancellous bone, is the proximal tibia. High tibial osteotomies are performed in the proximal tibia. Since the advent of locked plates for high tibial osteotomy, the osteotomy defect is not filled. In a study [14] published on this subject, the authors reported good results. The osteotomy gap healed in 91 of the 92 included patients in this study without augmentation of the osteotomy defect.

One questions why hand and wrist surgeons still use bone graft or bone substitutes for correction osteotomies in the distal radius while surgeons performing correction osteotomy on the proximal end of the tibia find them unnecessary.

In 2005, Wieland et al. [11] described a series of 47 patients where they performed correction osteotomy in the distal radius without filling of the osteotomy gap. They observed that all osteotomy gaps healed within 3 months. Furthermore, they found that leaving the gap empty, simplified the procedure substantially. However, their group was heterogeneous with inclusion of volar and dorsal displaced fractures and stabilisation was with dorsal or volar plates. Notably, they stabilised the osteotomy with conventional plates. Distal radius osteotomy and stabilisation with conventional plates leads to non-union occasionally, probably due to the poorer stability these plates provide [12].

Ozer et al. [9] described a case–control study of 14 distal radius osteotomies stabilised by a locked volar plate, without filling of the osteotomy gap, and found no delay in healing compared with the control group.

Our study is the first to describe correction osteotomies of the distal radius for dorsal malunion, with double dorsal plating, without filling of the osteotomy defect. With the latter, surgical—and tourniquet time was reduced. The patient did not have to undergo a bone-grafting procedure. Nor were bone substitutes or bioactive materials, which increase the costs for this procedure substantially, used. Despite this, our functional and radiological results were similar if not better than comparable series where bone grafting or bone substitutes were used.

The limitations of this study include factors relating to the retrospective nature of this study. Preoperative functional outcome scores were not measured, and there was no control group.

Another important limitation is the small number of patients included in this study. Even though the numbers are small, these eleven patients show that it is possible to promote union in the osteotomy gap of the distal radius without filling of the gap when locked dorsal plates are used.

Furthermore, since our primary endpoint was union, our follow-up time was relatively short to report reliably on complications related to this procedure such as osteoarthritic changes, tendon irritation or tendon ruptures. To measure the time saved and the effect limiting the surgery has on the adequate correction of the anatomy, a prospective study would be necessary.

In conclusion, performing distal radial correction osteotomy for dorsally displaced distal radius malunion, stabilised by dorsal bicolumnar locked plates can be performed safely in most patients with symptomatic malunion of the distal radius without filling of the osteotomy gap. The latter reduces surgical trauma and the operating time and simplifies the procedure. The surgeon can then concentrate on the most important part of the procedure: adequate correction of the deformity.
